# BayesPI - a new model to study protein-DNA interactions: a case study of condition-specific protein binding parameters for Yeast transcription factors

**DOI:** 10.1186/1471-2105-10-345

**Published:** 2009-10-20

**Authors:** Junbai Wang, Morigen

**Affiliations:** 1 Division of Pathology, The Norwegian Radium Hospital, Rikshospitalet University Hospital, Montebello 0310 Oslo, Norway; 2 Department of Cell Biology, Institute for Cancer Research, The Norwegian Radium Hospital, Rikshospitalet University Hospital, Montebello 0310 Oslo, Norway

## Abstract

**Background:**

We have incorporated Bayesian model regularization with biophysical modeling of protein-DNA interactions, and of genome-wide nucleosome positioning to study protein-DNA interactions, using a high-throughput dataset. The newly developed method (BayesPI) includes the estimation of a transcription factor (TF) binding energy matrices, the computation of binding affinity of a TF target site and the corresponding chemical potential.

**Results:**

The method was successfully tested on synthetic ChIP-chip datasets, real yeast ChIP-chip experiments. Subsequently, it was used to estimate condition-specific and species-specific protein-DNA interaction for several yeast TFs.

**Conclusion:**

The results revealed that the modification of the protein binding parameters and the variation of the individual nucleotide affinity in either recognition or flanking sequences occurred under different stresses and in different species. The findings suggest that such modifications may be adaptive and play roles in the formation of the environment-specific binding patterns of yeast TFs and in the divergence of TF binding sites across the related yeast species.

## Background

Regulation of gene expression is performed on several levels: transcription (DNA→RNA), translation (RNA→protein) and the post-translational modifications. Gene transcription is usually controlled by the interaction between regulatory factors and a regulatory DNA sequence located mostly on the up-stream of the transcription starting site. This regulatory region contains a short DNA sequence to which the gene regulatory proteins, such as transcription factors (TFs), bind in order to activate/repress the gene expression[1,2]. The DNA of eukaryotic cells is packaged into nucleosomes which build up chromatin. The DNA in the nucleosomes is not as readily accessible to binding of proteins like transcription factors as in naked DNA. The DNA packaging, nucleosome positioning and remodeling have been suggested to be mechanisms to control protein-DNA interactions involved in the transcription, replication and recombination [3]. The nucleosome positioning is coordinated by complex processes such as DNA-DNA interaction, protein-DNA interaction, histone modification, and chromatin remodeling[4]. Recent genome-wide experiments have generated high resolution genomic map of nucleosome locations in multiple species including yeast[5], *Drosophila*[6] and humans[7]. A few computational methods[8,9] have been used to investigate chromatin dynamics.

For silicon identification of the transcription factor binding sites, a pioneering work by Bussemaker *et al*. [10] suggests a linear regression model to infer the binding motifs of TFs on the regulatory region by combining high-throughput microarray data with DNA sequence information. Recent research in the field is integrating the biophysical model with the computational identification of TF binding sites [11-14]. Though the statistical-mechanical theory of selection of the DNA binding sites has been used for almost two decades[15], the application of such an old theory in a new way (computational prediction of TF binding sites using ChIP-chip dataset[16]) has generated numerous new algorithms. Most of these early developed methods are based on an assumption of low protein concentration, i.e., only the strongest DNA binding sites are bound by proteins. Thus, the protein binding probability in the models, namely MatrixReduce[12], MARS[17], PREGO[18], Precise Physical Models[13], BART[19] and CSI-Tree[20], is approximated by Maxwell-Boltzmann probability. The protein binding probability is dependent on the average number of proteins in a cell [15,21]. For a full biophysical modeling of protein-DNA interaction without the low protein concentration assumption, a term called chemical potential has been introduced[15]. The chemical potential is equivalent to the concentration of each protein inside a cell and can be changed when the protein binding probability varies in the cell [22,23]. Fermi-Dirac form of protein binding probability suggests that a DNA sequence with binding energy far below the chemical potential is always bound to a protein. In contrary, if the binding energy is well above the chemical potential then the sequence is rarely bound[21]. To build a full biophysical model for identifying protein binding sites using a high-throughput microarray dataset, a novel computational approach by incorporating the chemical potential with the protein binding probability is developed.

Except for the protein binding probability issue mentioned above, an improvement of the parameter fitting for a protein binding probability may also increase the accuracy of *in silico *prediction of protein-DNA interactions. Usually, the parameter estimation (the position-specific energy matrices of TF binding sites) is a nonlinear optimization problem[18]. An exact model fitting of such nonlinear parameters by using a regression method is not possible[18,24], which may suffer from "over-learning"[25]. Though the nonlinear parameter fitting is an important issue in designing a computer algorithm, it has rarely been addressed before[12,13]. We could use "cross-validation" and "early stopping" roles to partially overcome the obstacle. However, the best solution to deal with the nonlinear parameter fitting is a Bayesian method (i.e., Bayesian model regularization) [25] which has been proven to be a robust and comprehensible procedure to search for models that are better matched to the data.

We developed a new approach, a combination of Bayesian parameter inference, Fermi-Dirac form of protein binding probability and genome-wide positioning of nucleosome. The real value of our new program - BayesPI - was assessed on synthetic datasets and genome-wide ChIP-chip data. We also investigated the condition-dependent modification of protein binding energy matrices as well as the protein binding parameters (minimal TF binding energy and the corresponding TF concentration) by using the proposed new method. In earlier works, the protein binding parameters were seldom studied [22,26]. Particularly, few researchers have taken the advantage of using genome-wide TF occupancy data [27,28] to investigate either condition-specific or species-specific protein binding parameters. Through a systemic study of the protein binding parameters under different experimental conditions or across several related species, we may uncover certain crucial mechanisms behind the complex genome expression and regulation [29,30].

## Results

### Validation of BayesPI using synthetic ChIP-chip datasets

**Figure 1:**
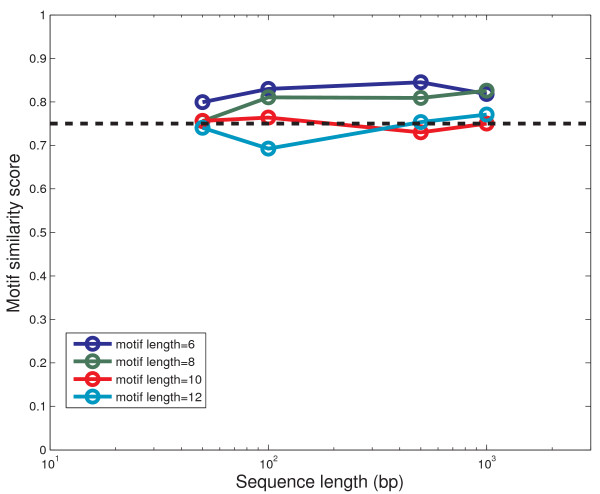
**Validation of the BayesPI using synthetic ChIP-chip datasets**. Here 16 synthetic ChIP-chip datasets were used in the program test and implanted motif lengths were 6, 8, 10 and 12 bp as indicated. The synthetic log ChIP-chip ratios follow a normal distribution [see Additional file 1: Supplemental Figure S1].

For an initial trial, we tested BayesPI on 16 synthetic ChIP-chip datasets where various lengths of binding motifs were implanted in synthetic DNA sequences. In Figure 1, we illustrated the results of these tests by a scatter plot of the motif similarity score as a function of the sequence length. The result indicates that the performance of BayesPI is quite robust against either the sequence length or the target motif size. All motif similarity scores are between 0.75 and 0.85, except for two cases with smaller motif similarity scores than the others. In these two cases, we found that the target motifs were long (i.e., motif length > 10 bp), suggesting that the long binding site may severely hinder the computational prediction of motif targets. To estimate good or bad motif matches, we identified a threshold value for the motif similarity score. For example, a similarity score > 0.75 represents a reasonable match between a pair of motifs. A score < 0.75 suggests a poor match [see Additional file 1: Supplemental Figure S2]. Such a threshold value, which has been used in a previous work [31], will be applied to compare the motif similarities among various predictions in follow-up studies.

### Validation of the BayesPI using real ChIP-chip datasets

After success on the synthetic datasets, we applied BayesPI on the real protein-DNA interaction datasets from ChIP-chip experiments in rich medium conditions [27]. We applied the same search strategy used in the synthetic datasets to find putative TF binding energy matrix for 61 selected yeast TFs. By comparing the putative TF binding energy matrix of each TF with the corresponding SGD (Saccharomyces Genome Database) consensus sequences, we found that ~88.5% of our predicted TF energy matrices had motif similarity scores greater than 0.75. The result demonstrates that the majority of our defined putative TF binding energy matrices (54 out of 61 TFs) bear good resemblance to the "known" SGD motifs.

MatrixREDUCE[12], a statistical mechanical model for genome-wide occupancy data, was used to compute the sequence specificities of 203 yeast transcription factor binding sites in rich medium conditions. These predicted position-specific affinity matrices (PSAM) were published in an online database, TransfactomeDB[32]. In the theoretical background, MatrixREDUCE and BayesPI share several similarities. They, however, have two major differences: i) the earlier approach does not use Bayesian method to control the fitting of model parameters by using least squares; ii) the protein binding probability of MatrixREDUCE is approximated by a Maxwell-Boltzmann distribution with an assumption of very low protein concentration, but such an assumption in BayesPI is lifted[15,21,22]. Therefore, a comparison between the TransfactomeDB PSAM and the SGD consensus sequences may provide insights on improving the model if we utilize a Fermi-Dirac form of protein binding probability to interrogate the same TF occupancy data[27]. Among all available TFs in the TransfactomeDB, we only compared 61 of them (e.g. the same 61 TFs used in the validation of BayesPI) with SGD consensus sequences. The result showed that there were ~50% good matches between the TransfactomeDB PSAM and the SGD consensus sequences. In other words, BayesPI did improve the prediction accuracy by considering both Bayesian inference and the Fermi-Dirac form of protein binding probability [15].

A number of binding sequence specificity information for DNA-binding proteins has been published. In particular, the sequence specificity data for yeast, TRANSFAC[33] and a publication by MacIsaac *et al*.[34] have been extensively used. The former is a manually created database that contains experimentally measured position weight matrices for particular DNA-binding proteins. The later is a recent refinement of yeast regulatory sites using two conservation-based motif discovery algorithms to reanalyze the genome-wide ChIP-chip data [27]. The sequence specificity information of 61 yeast TFs examined in this work are available in the collection from MacIsaac *et al*. (33), but that of 21 TFs are found in the TRANSFAC database. Comparisons of the two sequence specificity datasets with the present gold standard (SGD consensus sequences) showed that the percentage of motif similarity scores greater than 0.75 was ~87% for MacIsaac *et al*. and ~100% for TRANSFAC, respectively. This suggests that the quality of our predicted position-specific energy matrices using BayesPI is as good as the two most popular yeast sequence specificity repositories.

### Prediction of protein binding energy matrices by taking nucleosome positioning in consideration

As mentioned earlier, nucleosome occupancy affects transcription by decreasing the accessibility of DNA to protein binding. Thus, including nucleosome density may improve computational identification of protein binding sites using *in vivo *protein-DNA interaction datasets. Based on a modified protein binding probability (equation [8]), we tested BayesPI on a set of ChIP-chip experiments on 10 yeast TFs under rich medium conditions. It has been shown that three (MET31, RFX1 and PDR1) of the 10 TFs are not functional while the other seven are active under the growth conditions. Interestingly, we found that inferred protein binding energy matrices of the three non-functional TFs were poor [see Additional file 1: Supplemental Table S2], even if the binding of proteins is associated with the nucleosome-depleted region (i.e., PDR1[35]) and the nucleosome positioning information is considered in the program. Of the seven active TFs, five (SWI4, ACE2, MBP1, LEU3 and MCM1) showed either improved rank orders or increased motif similarity scores for the best motif when the nucleosome weighted protein binding probability was used, in which both SWI4-bound and LEU3-bound *loci *are known nucleosome-poor[8,35]. Experimental work has suggested that the low nucleosome occupancy at *loci *bound by LEU3 is not a consequence of LEU3 binding[8]. Two TFs, RAP1 and ABF1 are global regulatory proteins and can open chromatin in the vicinity of their binding sites [5,35-38], and did not show major improvements in the quality of their inferred binding energy matrices [see Additional file 1: Supplemental Table S2] although both TFs are known to bind nucleosome-depleted promoters. These outcomes indicate that the inclusion of the nucleosome information in the computation does improve the performance of BayesPI. However, the improvement is TF dependent because the activities of different TFs in the transcription are affected by the nucleosome position differently. It is to be noted that the effect of the inclusion of the nucleosome positioning information *in silico *motif prediction was not strong if a TF (i.e. PDR1) is non-functional under certain growth conditions.

### Protein binding parameters in the BayesPI prediction and other *in silico *calculations for a set of 61 yeast TFs

**Figure 2:**
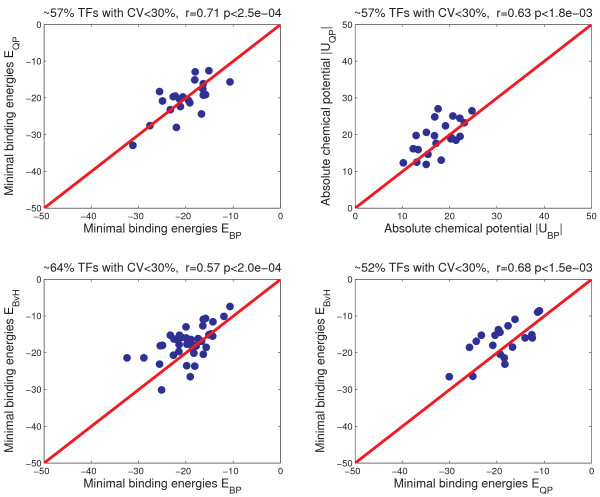
**Comparisons of the binding parameters for a set of 61 TFs of the yeast *S. cerevisiae *(YPD condition)**. *E*_*BP *_and *U*_*BP *_are minimal binding energy (consensus) and chemical potential estimated by the BayesPI, *E*_*QP *_and *U*_*QP *_are minimal binding energy (consensus) and chemical potential obtained by QPMEME[21], and *E*_*BvH *_is the minimal binding energy (consensus) computed from BvH[15]. CV is a coefficient of variation of the predicted binding parameters between two different methods. Here for each TF, if the CV<30% then we consider the two estimates are reasonably matched.

We have shown that the TF binding energy matrices derived from BayesPI are consistent with the known sequence specificities. The new method is robust on both synthetic simulated datasets and real TF occupancy datasets. However, there are several protein binding parameters (TF minimal binding energy and the corresponding chemical potential) need to be verified. Aurell *et al*. [26] calculated transcription factor concentrations (chemical potential) and the corresponding minimal binding energies (consensus) for 61 yeast TFs in rich medium conditions. They used two separate methods, the classical work of Berg and von Hippel [15] (BvH) and an approximation of recently introduced Quadratic Programming Method of Energy Matrix Estimation [21] (QPMEME or QP), to estimate the minimal binding energy of a specific TF. BvH does not include chemical potential. QPMEME may estimate the chemical potential but it fails if the measured TF abundance [39]*n*_*obs *_is too low. As shown in [Additional file 1: Supplemental Table S1], both BayesPI (BP) and BvH gave solutions to all 61 TFs in rich medium conditions but QPMEME only solved 38 of them due to the internal limitation of the method [26]. A comparison between BP and QPMEME shows that ~57% of estimated minimal energies (consensus, p < 2.5e-4) and ~57% of predicted chemical potential (p < 1.8e-3) have a reasonable match. Following the same threshold [see Additional file 1], a comparison between BP and BvH reveals that ~64% of predicted minimal binding energies (p < 2.0e-4) have a good match. However, a comparison between QPMEME and BvH shows only ~52% of the predicted minimal energies (p < 1.5e-3) have good correlations. Thus, the estimation by BP is more consistent with the BvH prediction than the QPMEME estimation. This may be explained by the similarity of the biophysical background behind both BP and BvH methods[15]. Figure 2 shows scatter plots of the above comparisons across three methods. The results indicate that the estimated protein binding parameters of 61 yeast TFs in rich medium conditions are comparable among three different calculations (BP, BvH and QPMEME).

### Computation of condition-specific protein binding parameters for yeast TFs

**Figure 3:**
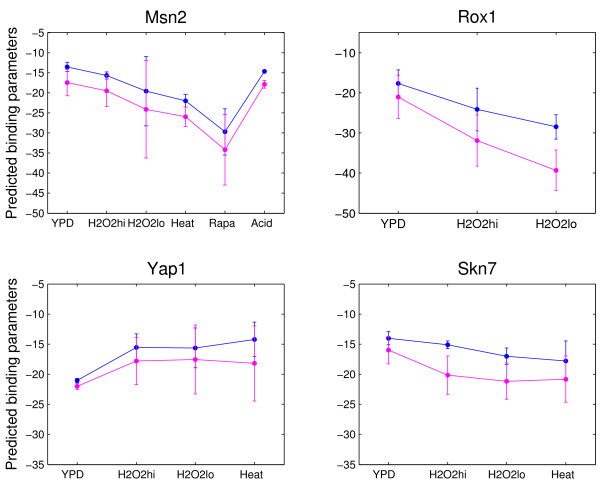
**Condition-specific binding parameters for four yeast TFs known for being activated by stress conditions**. Y-axis are predicted TF binding parameters (e.g. minimum binding energy and chemical potential) by the BayesPI. Magenta line stands for minimal binding energy *E*_*BP *_(consensus) and blue line for chemical potential *U*_*BP*_. For estimating uncertainties in the predictions, error bars of binding parameters were computed by applying the BayesPI three times on each ChIP-chip dataset, 80 percent of randomly selected original dataset was included in every calculation.

As shown above, the TF binding energy matrices computed by BP are consistent with the SGD consensus sequences. Particularly, the calculated TF binding parameters [see Additional file 1: Supplemental Table S1] by BP are in line with the other calculations in yeast under rich medium conditions (YPD). Thus, we could apply the new method to investigate condition-dependent TF binding parameters, i.e., stress-specific protein binding parameters. Here we selected four yeast TFs (i.e., MSN2, ROX1, YAP1 and SKN7) from *in vivo *ChIP-chip experiments[27]. These TFs are known to be required for activation of the stress-induced gene transcription. For example, (1) MSN2, a zinc finger protein, is a transcriptional activator of the multi-stress responses in *S. cerevisiae *[40] such as heat shock (heat), oxidative stress (H_2_O_2_), glucose starvation (Rapa) and sorbic acid (Acid) [40]. Our estimation in Figure 3 shows that the chemical potential and minimal binding energy of MSN2 (large negative value represents high chemical potential or binding energy [41]) were increased due to H_2_O_2_, heat and Rapa but not with acid. (2) ROX1, a heme-dependent repressor of hypoxic genes, may repress its own expression by binding its own upstream region to prevent the accumulation of excess ROX1 in the cell under high oxygen levels[42,43]. Here we found an increasing of ROX1 binding parameters under hyperoxic conditions (H_2_O_2_) relative to the YPD. Such increasing to high hyperoxic condition (H_2_O_2 _hi) was relatively smaller than that to low hyperoxic condition (H_2_O_2_lo) (Figure 3). The result may reflect a self-repression role of ROX1 to control its own cellular level [43]. (3) YAP1 [44] and SKN7 [45] are essential for resistance to oxidative stress (H_2_O_2_) response but do not always function together in the activation of H_2_O_2_-inducible genes [46]. Here we observed that the minimal binding energy and chemical potential for YAP1 were decreasing while those for SKN7 were increasing under heat and hyperoxic conditions although the responses were weak (Figure 3). These results are in agreement with previous reports which found YAP1 and SKN7 cooperatively control several H_2_O_2 _target genes, exerting the same or opposite effects[46]. It is worth noting that the protein binding parameters for each TF investigated responded in essentially the same pattern to each stress.

**Figure 4:**
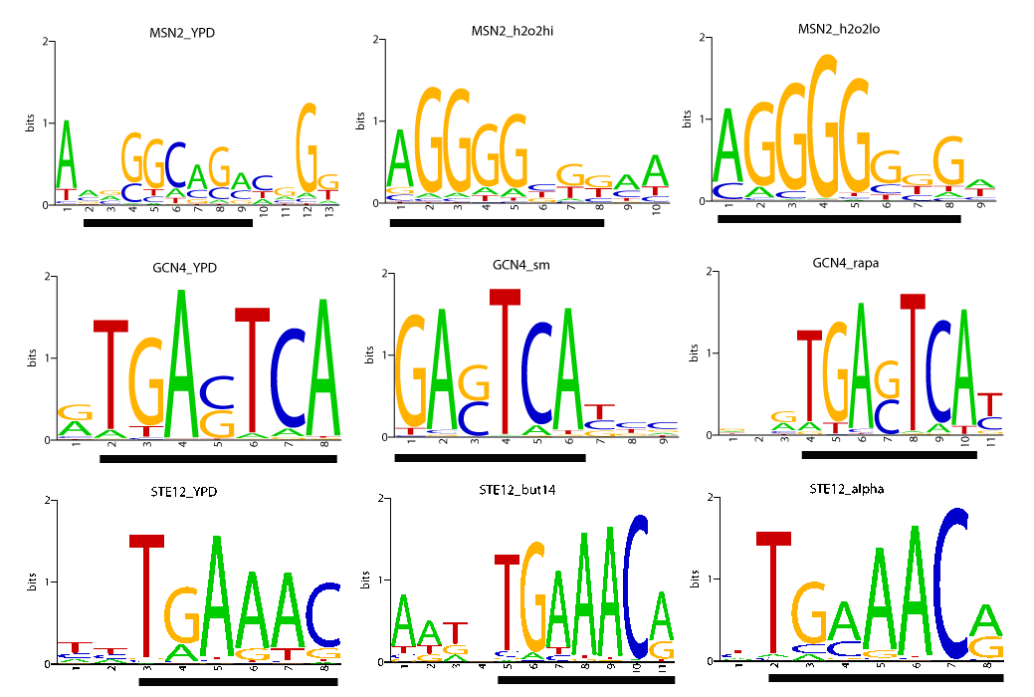
**Condition-specific binding energy matrices for three yeast TFs**. Condition enabled binding -- MSN2 binding site (AGGGGSGG), condition expanded binding -- GCN4 binding site (TGASTCA) and condition altered binding -- STE12 binding site (TGAAACR) [27] were included. The sequence logo was generated by energy matrices estimated by the BayesPI.

In a recent work, Harbison *et al*[27] had characterized several types of environment-specific binding for yeast transcriptional regulators: condition-invariant, condition-enabled, condition-expanded, and condition-altered binding behaviors. We are particularly interested in the last three because these binding events are dynamically changed during a shift of a condition. For example, the stress response factor MSN2 belongs to a condition-enabled binding as shown above (Figure 3). We have used the BP method to estimate the protein binding energy matrices (Figure 4) and the corresponding binding parameters (Table 1) for the last three types of condition-specific bindings. Here is an overview of the results: i) for condition-enabled binding protein MSN2 in rich medium condition, we found a marginal match between the inferred binding energy matrices and the SGD consensus sequence (motif similarity score ~0.76; Table 1). However, under the oxidative stress known as an activation condition (H_2_O_2_lo and H_2_O_2_hi) for MSN2, we found the inferred binding energy matrices changed in both binding parameters (i.e. ~17.4% and ~22.8% variation[47,48] in predicted chemical potential in H_2_O_2_lo and H_2_O_2_hi, respectively, compared to that under the YPD condition, Table 2) and shape (Figure 4) but still had good resemblance to the "known" consensus sequence (i.e. motif similarity score ~0.91 and ~0.92 for H_2_O_2_lo and H_2_O_2_hi, respectively; Table 1); ii) For condition-expanded binding factor GCN4, under different conditions (YPD, amino acid starvation (Sm), and nutrient deprived (Rapa)), the binding energy matrices were similar to each other (Figure 4) as well as to the SGD consensus sequence (motif similarity score ~0.94 for YPD, ~0.93 for Sm, and ~0.94 for Rapa, respectively; Table 1). Particularly, the predicted binding parameters under different conditions did not show any dramatic changes (~1.7% variation[47,48] for Sm and ~2.9% for Rapa in minimal binding energy, Table 2); iii) For condition-altered regulators STE12 under different conditions, we not only observed a strong variation of the shape of TF binding energy matrices (Figure 4) but also found a clear alteration of the corresponding binding parameters (~16% for Alpha and ~30% for But14 in the chemical potential; ~15% for Alpha and 40% variation[47,48] for But14 in the minimal binding energy relative to these in YPD; Table 2), though motif similarity scores were all good (~0.91, ~0.92, and ~0.89 for YPD, But14 and Alpha, respectively, Table 1).

**Table 1:** Condition-specific binding parameters for three yeast TFs.

**Binding behavior**	**TF name**	**Environment**	***E*_*BP*_**	***U*_*BP*_**	**Motif similarity score**
**Condition enabled**	**MSN2**	**YPD**	-21.96	-13.97	**0.76**
	**MSN2**	**H_2_O_2_hi**	-18.43	-17.57	0.92
	**MSN2**	**H_2_O_2_lo**	-21.54	-16.64	0.91

**Condition expanded**	**GCN4**	**YPD**	-23.84	-22.73	0.94
	**GCN4**	**Sm**	-24.26	-21.68	0.93
	**GCN4**	**Rapa**	-24.54	-21.27	0.94

**Condition altered**	**STE12**	**YPD**	-17.80	-16.40	0.91
	**STE12**	**But14**	-26.45	-22.14	0.92
	**STE12**	**Alpha**	-20.71	-19.25	0.89

Minimal binding energy (consensus) *E*_*BP *_and chemical potential *U*_*BP *_are estimated by the BayesPI using ChIP-chip experimental data under various conditions [27], such as rich medium condition (YPD), a high hyperoxic (H_2_O_2_hi), a moderate hyperoxic (H_2_O_2_lo), amino acid starvation (Sm), nutrient deprived (Rapa), filamentation inducing (But14) and mate inducing (Alpha).

**Table 2:** Statistical analysis for accessing difference of the predicted yeast protein binding parameters between paired conditions.

	**Condition enabled TF - MSN2**		**Condition expanded TF - GCN4**		**Condition altered TF - STE12**	
	
	**YPD vs. H_2_O_2_hi**	**YPD vs. H_2_O_2_lo**	**YPD vs. Sm**	**YPD vs. Rapa**	**YPD vs. But14**	**YPD vs. Alpha**
**|ΔE|**	17.5%	2.0%	1.7%	2.9%	39.1%	15.1%
**|Δμ|**	22.8%	17.4%	4.7%	6.7%	29.8%	16.0%

The description of each condition is similar to that in Table 1. The |ΔE| or |Δμ| is the absolute difference between the paired minimal binding energies or chemical potential (*E*_*BP *_or *U*_*BP *_in Table 1) divided by their mean minimal binding energy or mean chemical potential, respectively.

Considering these results together, we hypothesize that for condition-enabled regulators there is a clear difference between *to bind *(active) and *not to bind *(not active) via sequence specificity once the binding condition is reached. However, for condition-altered regulators the variation of sequence specificity between different conditions is less distinguishable. In other words, condition-altered bindings may share close sequence specificity among various conditions but the binding affinity of each individual binding site may have position-specific variation, for example, dimers TG and CA in the STE12 binding sites (Figure 4). Nevertheless, condition-expanded regulators not only share the same sequence specificity among different conditions but also keep similar binding parameters in various environments such as GCN4 (Tables 1 and 2). Thus, we speculate that the condition-altered regulators (i.e., STE12) may represent a set of the most active TFs in a cell since they regulate a large number of diverse set of genes under different conditions by cooperating with many other transcription regulators[49]. Such diverse binding activities of condition-altered regulators may benefit from the great flexibility of their binding parameters (i.e., an adaptive change of binding affinity in an individual nucleotide [50]).

### Estimation of species-specific protein binding parameters for yeast TFs

So far we have characterized the property of the environment-dependent transcription factor binding parameters for several yeast(*S. cerevisiae*) TFs. The results suggest that both TF binding parameters and the corresponding motif binding energy matrices may undergo environment-specific modifications. It is interesting to see whether such modification of protein binding parameters would be in a species-specific manner. For that reason, we obtained ChIP-chip datasets and the relevant DNA sequence information from an investigation on STE12 and TEC1 binding sties across three related yeast species (i.e., *S. cerevisiae *- Scer, *S. mikatae *- Smik, and *S. bayanus*- Sbay) under the same pseudohyphal conditions [28]. Here we used the BP method to compute both binding energy matrices and binding parameters for the above two TFs across the three species. The estimated binding parameters of each TF for each replicated ChIP-chip experiment for each yeast species are listed in Table 3. We found that at least two of the triplicate experiments for either STE12 or TEC1 showed similar binding parameters in both Smik and Sbay, which suggests a good reproducibility of the predictions (Table 3). The recovered binding energy matrices of the three experiments in each species also showed similarity [see Additional file 1: Supplemental Figures S4 and S5]. Especially, motif similarity scores were good for all 16 ChIP-chip experiments, except for one case where the motif similarity score was less than 0.75. Thus, the sequence specificity for either STE12 or TEC1 does share similarities across the three species but the protein binding parameters bear a species-specific variation.

**Table 3:** Species-specific binding parameters for two yeast TFs.

**TF**	**Species**	**Experiment design**	***E*_*BP*_**	***U*_*BP*_**	**Motif similarity score**	**Median *E*_*BP*_**	**Median *U*_*BP*_**
STE12	Scer	R	-18.1	-15.09	0.82	-18.31	-15.09
				
		R	-35.86	-18.16	**0.72**		
	
	Smik	R	-24.40	-21.11	0.91	-17.35	-17.17
				
		R	-17.35	-16.44	0.90		
				
		D	-16.96	-17.17	0.90		
	
	Sbay	R	-16.52	-16.24	0.86	-16.52	-16.24
				
		R	-16.47	-15.7	0.86		
				
		D	-21.72	-18.02	0.91		

TEC1	Scer	R	-15.63	-15.46	0.91	-18.66	-15.96
				
		R	-21.68	-16.45	0.92		
	
	Smik	R	-11.82	-12.95	0.90	-14.03	-13.59
				
		R	-15.92	-15.88	0.91		
				
		D	-14.03	-13.59	0.90		
	
	Sbay	R	-19.39	-13.43	**0.79**	-15.46	-13.57
				
		R	-14.95	-12.63	0.83		
				
		D	-15.97	-14.50	0.93		

Minimal binding energy (consensus) *E*_*BP *_and chemical potential *U*_*BP *_are estimated by the BayesPI based on ChIP-chip experimental data from *S. cerevisiae *(Scer), *S. mikatae *(Smik), and *S. bayanus *(Sbay) under pseudohyphal conditions. R represents replicated experiment, and D means dye-swapped experiment.

**Figure 5:**
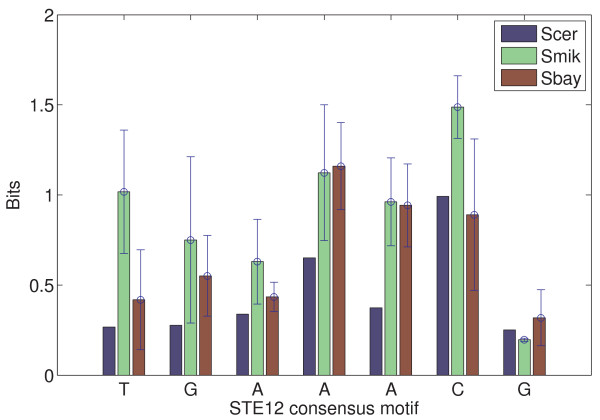
**Comparison of STE12 binding energy matrices in three yeast species: *S. cerevisiae *(Scer), *S. mikatae *(Smik), and *S. bayanus *(Sbay)**. Only the information content (binding affinity was transformed to bits) at every position in a STE12 consensus sequence (TGAAACG) is shown. The height of each bar is the mean information content at each position that was estimated from replicated ChIP-chip experiments, and the error bar is the standard deviation of these estimates. Sequence logo representations of the STE12 energy matrices are available in [Additional file 1: Supplemental Figure S4].

**Figure 6:**
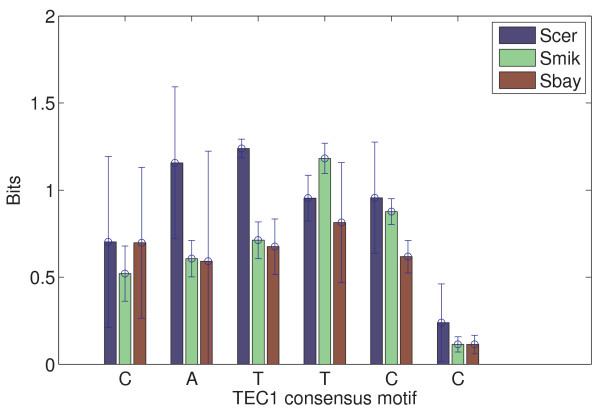
**Comparison of TEC1 binding energy matrices in three yeast species: *S. cerevisiae *(Scer), *S. mikatae *(Smik), and *S. bayanus *(Sbay)**. Only the information content (binding affinity was transformed to bits) at every position in a TEC1 consensus sequence (CATTCC) is shown. The height of each bar is the mean information content at each position that was estimated from replicated ChIP-chip experiments, and the error bar is the standard deviation of these estimates. Sequence logo representations of the TEC1 energy matrices are available in [Additional file 1: Supplemental Figure S5].

Table 4, for STE12, displays a 13% variation[47,48] of the chemical potential (or 10% in the minimal binding energy) between Scer and Smik (or Sbay) but it only gives about 5% difference between Smik and Sbay; for TEC1, the variation of the binding parameters between Scer and Smik (or Sbay) was greater than 16% but it was less than 10% between Smik and Sbay. These results suggest that closely-related species (i.e., Smik and Sbay[28]) bear less variation in the protein binding parameters. Particularly, the shape of the predicted position-specific energy matrix of each TF also varies across the three species [see Additional file 1: Supplemental Figures S4 and S5]. For example, the magnitude of protein binding affinities of a few key binding positions shows species-specific variation (Figures 5 and 6): binding affinities of the pyrimidine-purine dimers YR (TG:CA) and the AA dimer in the STE12 binding site (TGAAACR) [see Additional file 1: Supplemental Figure S4], and another two dimers (CA and TT) in the TEC1 binding site (CATTCY), as well as a nucleotide in the flanking sequence (the nucleotides on the right side of TEC1 binding site; [see Additional file 1: Supplemental Figure S5]) were found to be modified. The mechanism behind such species-specific variation of individual nucleotide affinity seems similar to the stress-dependent alteration. It is possible that affinities of a few important protein binding positions are being adaptively changed under environment perturbation. Thus, a combination of the adaptive modification of protein binding parameters and the conditional variation of protein binding affinities in DNA sequences may play roles in divergence of TF binding sites across the three yeast species.

**Table 4:** Statistical analysis for accessing difference of the predicted protein binding parameters between paired yeast species.

	**STE12**			**TEC1**		
	
	**Scer vs. Smik**	**Scer vs. Sbay**	**Smik vs. Sbay**	**Scer vs. Smik**	**Scer vs. Sbay**	**Smik vs. Sbay**
**|ΔE|**	5.4%	10.3%	4.9%	28.3%	18.7%	9.7%
**|Δμ|**	12.9%	7.4%	5.6%	16.0%	16.2%	0.2%

The description of each species is similar to that in Table 3. The |ΔE| or |Δμ| is the absolute difference between the paired minimal binding energies or chemical potential (*E*_*BP *_or *U*_*BP *_in the Table 3) divided by their mean minimal binding energy or mean chemical potential, respectively. The predicted protein binding parameters with motif similarity score < 0.8 was not considered in the comparison.

## Discussion and Conclusion

In this work, we have developed a new computational method, BayesPI (BP), to study protein-DNA interaction by using high-throughput yeast ChIP-chip microarray. Validation of the BP method on both synthetic datasets and real experimental data shows that BP is robust. Comparisons of protein binding parameters (e.g., minimal protein binding energy and the corresponding chemical potential) of 61 yeast TFs obtained by BP with the estimations from previous models give good correlations ([see Additional file 1: Supplemental Table S1] and Figure 2). In particular, the newly introduced protein binding parameters in the BP method provide an extra dimension to explore the complex transcription regulation. For example, the method allows us to observe the change of protein binding energy matrices under different conditions, and the modification of both protein binding energy and associated chemical potential after environmental perturbations (Figure 3, 4 and Tables 1 and 2). The elasticity of the protein binding parameters under different environments is also found to be true in a species-specific manner (Figure 5 and 6, and Table 3 and 4) which may be used to judge the phylogenetical distance among species (Table 4). Particularly, we noticed the position-specific variation of protein binding affinities at a few key binding sites when being exposed either to a new environment or to another species. For example, the pyrimidine-purine dimers YR (TG:CA) in the STE12 consensus are the most flexible nucleotides with respect to the binding affinity changes. An early DNA sequence-dependent deformability study from protein-DNA crystal complexes also suggests the same YR dimers are the most flexible base pairs on the DNA sequences[51]. Such sequence-dependent structural effects in the DNA duplexes may indicate that certain nucleotides in the DNA are more flexible and influenced by their surroundings [52,53].

In addition to the variation of the individual nucleotide affinity in protein binding sites, we also observed a similar modification in the flanking sequence such as the nucleotide adjacent to the right side of TEC1 binding site C, T, and G in Scer, Smik and Sbay, respectively [see Additional file 1: Supplemental Figure S5]. This is in line with a previous proposal of that the flanking sequence variation may affect the energy required for DNA distortion, the binding affinity of the nearby protein binding sites may be either increased or decreased, and consequently may influence protein-DNA regulation[50]. The significance of such flanking sequences variation for gene expression and regulations was also suggested in a few recent studies. For example, alternative transcriptional regulation by a2-MCM1 binding in different yeast species [54] and disruption of p53-MDM2 oscillation by SP1 binding in the human MDM2 promoter [55] have been reported. Therefore, the variation of nucleotide in either protein recognition sequence or flanking sites may result in a change in protein binding affinity [56] by responding to different conditions, which lead to distinct gene expression patterns[57]. This may also explain the adaptive modification of protein binding parameters in both environment-specific (Figure 3 and 4) and species-specific protein-DNA interactions (Figure 5 and 6). The newly introduced protein binding parameters are possible phenotypes of binding sites' evolution as well as of function and non-function protein binding sites.

The new approach, the Bayesian model regularization, has overcome an important obstacle in nonlinear parameter fitting: for example, both MatrixREDUCE[12] and Precise Physical Models[13] are required to rescale their results (i.e., the estimated protein binding energy matrix) to normalize the matrix elements range between 0 and 1. The rational of the rescaling of the energy matrix is vaguely explained in the earlier publications. However, we knew that the above problem is often caused by non-convergence (or overfitting) of nonlinear parameter fitting (i.e., if the algorithm cannot converge or overfit the data then very large matrix elements will appear). In such a case, previous methods have to rescale the parameters after the calculation is completed, but the Bayesian approach can control the parameters online by searching for model parameters that are better matched to the data[25]. The performance of the BayesPI is definitely improved by considering the Bayesian method.

Finally, the prediction of protein binding parameters using BayesPI is promising. However, the power of applying a biophysical model for protein-DNA interaction still has room for improvement. Here are three possible directions to make BayesPI more useful in the future: 1) for the current implementation of the Bayesian nonlinear parameter fitting, we used Gaussian approximation for probability distribution to estimate the model parameters. However, not all datasets are suited for the Gaussian approximation, thus, a Markov chain Monte Carlo sampling of probability distribution may be considered in the future[25]; 2) for the computation of protein binding energy matrix by using the biophysical model, we assumed that every base contributes independently to the protein binding, which means the total energy of the interaction is only the sum of the energies of the individual contact. Though such additive assumption may provide good approximation to the true nature of the protein-DNA interactions[58], experimental observations had recommended that the future computational methods should take into account the non-independence of bases in the nucleotides of the protein binding sites[59,60]. For example, a biophysical model with a pair-dependent correction of the energy matrix may give better description of the true binding site specificities[21]; 3) for the identification of the functional regulatory binding sites, the protein binding probability is not the only factor to define them; other variables including adjacent co-regulator binding sites, distance from the transcriptional start site, genome conservation and nucleosome positioning may affect the degree of function of a binding site. For these reasons, future algorithms should use sophisticated methods to include at least some of the additional variables discussed.

## Methods

### Prediction of TF binding parameters using BayesPI

#### Biophysical modeling of Protein-DNA interactions

In a general biophysical modeling [12] of protein-DNA interactions,

(1)

binding of a protein to DNA with reaction constants *k*_a _(protein-DNA association) and *k*_d _(protein-DNA dissociation) can be expressed as the quotients of the two rate constants above,

(2)

where *K *is an inverse equilibrium concentration, Δ*G*(*S*) is a standard Gibbs free energy exchange of a protein binding to a short stretch of DNA with sequence S, R is the gas constant and T is the absolute temperature. Any position in the sequence S that does contribute to the site-specificity binding will exhibit Δ*G*_*S*_(*b*)<0 for some subset of b, while the other base types at that position will exhibit Δ *G*_*S*_(*b*) > 0, *b *∈⟨*A*, *C*, *G*, *T*⟩ [41]. The probability [21] of the DNA sequence S to be bound by a protein in a solution with the concentration n_*protein *_is given by

(3)

thus

(4)

where *μ *is the chemical potential set by the protein concentration *μ *= *RT *ln(*Kn*_*protein*_).

To further simplify the Fermi-Dirac form of protein binding probability, we may assume that the concentration of protein is so low that all DNA sequences S have very low probability of being bound[21], in which  and the Fermi-Dirac function can be approximated by a Maxwell-Boltzmann function

(5)

This approximated protein binding probability was used by earlier works such as MatrixREDUCE[12] to model genome-wide TF occupancy data by combing ChIP-chip profiles and position-specific affinity matrices (PSAM) prediction for each TF,

(6)

where t_i _is the measured ChIP profiles and F, C, and Δ*G *are parameters that are estimated by least squares fitting.

#### Incorporating nucleosome density into the biophysical modeling of protein-DNA interactions

It has been shown that nucleosome occupancy plays a key role in the accessibility of DNA to transcription factors [8,9]. Thus, a model of DNA occupancy by transcription factors and nucleosomes, expressed in terms of probabilities, is likely to give a more complete view of the protein-DNA interactions. Based on this idea, we modified the *k*_d _values in the equation [3] to consider the effect of nucleosome occupancy in the protein binding region,

(7)

This weighted *k*_d _was first suggested by Liu *et al*. [8], but we are the first to attempt to incorporate this modified *k*_d_(weighted) into the protein binding probability P(S),

(8)

where *Nuc*_ocu _is the measured nucleosome occupancy in the genomic region spanning the site S, and *W *is a weighting parameter. In the original work [8], the authors had to manually adjust the parameter *W *to find the best value on their experimental data. However, in the present work, the *W *can be estimated automatically from the equation [6] using a novel Bayesian minimization method that will be introduced later.

#### Building of Bayesian minimization model

We briefly described the transformation of the biophysical modeling of TF-DNA interaction into a nonlinear minimization problem. In this transformation, the measured ChIP-chip profiles for each TF, genome-wide nucleosome occupancy data, and the predicted TF concentration with its binding free energy Δ*G *were combined. Now we will solve the minimization problem with a novel Bayesian method. First, the model error function can be written as

(9)

where t_i _is the measured ChIP-chip affinity profile to gene i: for example, we used all available raw ChIP-chip ratios for each TF in the present yeast *S. cerevisiae *study; and Y_i _is the predicted TF occupancy data for that gene according to the TF binding probability, such as equations [4] or [8],

(10)

In the above equation, *w *is the motif weight, b is the motif bias, and the protein binding probability can be expressed as

(11)

if equation [4] is used, in which *l *= 1...n-M+1 (n is the length of sequence length and M is the length of motif) and i = 1...g (g is the number of genes). In equation [11],  is equivalent to the TF binding energy matrices Δ*G *(for each TF, the sum of all negative  values is its minimal binding energy[41]), and *b' *represents the chemical potential μ. To avoid possible overfitting to the noise on the data t_i_, we added a penalty term, regularization term E_w_, to the error function [9]. Here a simple weight decay form of regularizer is used,

(12)

which consists of the sum of the squares of all Q parameters in the model such as *w*_*q *_∈ ⟨, *b'*, *w*, *b*⟩. By using a regularizer of the form [12], the parameters are encouraged to be small.

In a Bayesian framework, we consider a probability distribution over parameters of *w*. Before including any data, this is described by a prior distribution *P*(*w *| *α*, Λ, Γ), in which ⟨Λ,Γ⟩ represents the model hypothesis space (Λ is the definition of protein binding probability and Γ is the selection of a regularization function) and *α *is a hyperparameter that controls the distribution of all other parameters of *w*. Once we observe the data D, we can write down an expression for the posterior probability distribution for the parameters of *w*, denoted by *P*(*w*|*D*, *α*, *β*, Λ, *η*, Γ). *β *is another hyperparameter that controls the variance of the data noise, using Bayes' theorem

(13)

where *P*(*D *| *α, β*, Λ, Γ, *η*) is a normalization factor and the quantity *P*(*D *| *w*, *β*, Λ, *η*) represents a model for the noise process on the target data. In general [25,61], we can write both *P*(*w *| *α*, Λ, Γ) and *P*(*D *| *w*, *β*, Λ, *η*) distributions as an exponential form, for example

(14)

where *Z*_*w*_(*α*) is a normalization factor given by *Z*_*w*_(*α*) = ∫*dw*exp(-*αE*_*w*_); and

(15)

the function *Z*_*D*_(*β*) is also a normalization factor given by *Z*_*D*_(*β*) = ∫*dD*exp(-*βE*_*D*_). After we have defined both distributions in equations [14] and [15], we can use equation [13] to find the posterior distribution of the parameters

(16)

where

(17)

and *Z*_*M*_(*α, β*) = ∫*dw*exp(-*M*(*w*)). Here the task is to find the most probable parameters of *W*_*MP *_given the input data D and the hypothesis model space ⟨Λ, *η*, Γ⟩ based on equation [16]. This problem can be solved by using a Gaussian approximation for the posterior distribution [25] which will be introduced in the next section.

#### Parameter inference of Bayesian minimization model

Based on a probability framework of the objective function [17] described above, we use a two-level inference method [25] to learn the parameters such as *w*_*MP*_, *α,β*.

i) We assume that the values of α and β are known and then infer the model parameters of *w*_*MP *_through the posterior probability *P*(*w*|*D*, *α*, *β*, Λ, *η*, Γ). By Taylor-expanding and Gaussian approximation [25] the log posterior probability, we get

(18)

where *A *= -∇∇ log *P*(*w*|*D*, *α*, *β*, Λ, *η*, Γ). To maximize this log posterior probability, we obtained the derivative of the log posterior probability with respect to the model parameters of *w *and set the derivatives to zero [25]. Subsequently, we applied a scaled conjugate gradient algorithm [62] to find the most probable values for the parameters of *w*_*MP *_by the given input data and hypothesis space ⟨Λ, *η*, Γ⟩. More information is available in [Additional file 1] and other relevant references [25].

ii) We update model parameters of *w*_*MP *_then infer α and β through Bayes' rule:

(19)

where *P*(*α*, *β*|*D*, Λ, *η*, Γ) is the posterior probability of hyperparameters α and β given the input data D and hypothesis space ⟨Λ, *η*, Γ⟩, *P*(*D*|*α, β*, Λ, *η*, Γ) is the data-dependent evidence for hyperparameters α and β, and *P*(*α, β*|Λ, *η*, Γ) is the subjective prior over our hypothesis space. Here we assume equal priors *P*(*α*, *β *| Λ, η, Γ) to the alternative models and a constant term to the *P*(*D *| Λ, Γ, η) then the model [α,β] is ranked only by evaluating the evidence:

(20)

Thus the log evidence for hyperparameters α and β is

(21)

To find the condition that is satisfied at the maximum log evidence, we first need to differentiate the log evidence with respect to α or β then set the derivative to zero, which results in two conditions [25] suited for the most probable values of α and β:

(22)

(23)

where γ = *k *- *α*•*Trace*(*A*^-1^) and *A *= ∇∇*M *are the Hessian of M(w) evaluated at *w*_*MP*_, k is the dimension of parameters of w, and N is the degree of freedom in the data set, such as the number of genes.

#### Computation of Hessian matrices

The two conditions (equations [22] and [23]) described for hyperparameters α and β could be used as re-estimation formulas for the Bayesian model fitting. However, an important issue is how to evaluate the Hessian matrix **A**, which requires an efficient algorithm [63] (fast exact multiplication by the Hessian) to compute products **A*v ***without explicitly evaluating **A**, where ***v ***is an arbitrary row vector whose length equals the number of parameters in the model. To calculate **A*v***, we define a differential operator *R*{•} based on the R-propagation algorithm of Pearlmutter [63]

(24)

which infers *R*{∇_*w*_} = *Av *and *R*{*w*} = *v*. By applying the *R*{•} operator on the Back-propagation neural networks (more detailed information is given in [Additional file 1]), we can easily compute the Hessian matrix multiplied by an arbitrary vector *v*.

#### Implementation of the Bayesian minimization model - BayesPI

We encoded the two-level inference of Bayesian minimization model (Bayesian nonlinear parameters fitting) in a MATLAB toolbox, resulting in a novel method, BayesPI. Parts of the programs (scaled conjugate gradient algorithm, back-propagating learning procedure for neural networks, R-forward computation, and R-backward computation) have been developed in C that is an external program of the MATLAB environment. For building the neural network topologies and the evidence updating function in BayesPI, we used NETLAB toolbox[64]. The program can run in both Linux/Unix and Windows environments, and the source code is publicly available . In the present study, for one yeast genome-wide ChIP-chip experiment, BayesPI spends approximately 54 hours on a PC cluster (using a dual-core CPU SUN X6220 blade node, with 16 GB of RAM) to complete the calculation of top six binding energy matrices with 3 possible motif lengths (i.e., ranging from 8 to 10), respectively. Such heavy computational requirement could be significantly shortened if we parallelize the code (i.e., using MPI language) and run it in parallel.

### Prediction of TF binding parameters using the classical work of Berg and von Hippel (BvH)

BvH[15] computes the discrimination energy (the binding properties of the TFs) E for a certain DNA sequence  by equation

(25)

where λ is a proportionality factor to relate populations of base-pair choices to binding free energies and varies between ~0.5 and ~1.5 at most, *B*_*l *_is each possible base-pair B (B = 0, 1, 2, 3, where e.g. 0 = AT, 1 = TA, 2 = GC, and 3 = CG) at position l (l = 1, 2,...s, where s is the binding site size),  is the number of occurrences of base-pair B at position l in the sample of N sites, and *n*_*l*0 _is the number of occurrences of the strongest binder B at that position.

### Prediction of TF binding parameters using Quadratic Programming Method of Energy Matrix Estimation (QPMEME)

In QPMEME [21], the ratio r of binding energies to a chemical potential is calculated first, then the absolute chemical potential |*μ*| is estimated through below relationship

(26)

where *n*_*obs *_is the measured TF abundance from a previous work[39], L is a genomic sequence length in a cell, *ρ*(*r*) is the background density of states for the energy matrix E obtained from QPMEME, and β equals .

### Motif similarity score

In an *in silico *study of TF binding sites, we often encounter either the evaluation of the quality of the estimated sequence specificity or the identification of the original of predicted sequence specificity. Thus, a method is needed to compute the similarity between a predicted TF binding motif and a set of known sequence specificity information, such as the consensus sequences from the SGD database [65]. In this work, we used a published computational strategy (i.e., motif similarity score[66]; a detailed description is provided in [Additional file 1]) to accomplish the goal.

### Synthetic datasets

To test our newly developed BayesPI, we made a set of synthetic ChIP-chip datasets: i) four types of synthetic DNA sequences with various sequence lengths (i.e., 50 bp, 100 bp, 500 bp, and 1000 bp, respectively) were generated by Monte Carlo sampling method through the MATLAB Bay Net toolbox[67]; ii) the relevant synthetic ChIP-chip log ratios were produced by the MATLAB build-in random number generator; iii) four yeast TFs (i.e., ACE2p, SWI4p, INO4p and XBP1p with different binding motif length of 6 bp, 8 bp, 10 bp and 12 bp, respectively) were selected as potential prediction targets for the test; iv) for each synthetic ChIP-chip data, one of the corresponding TF binding motifs was randomly positioned in a DNA sequence in which the associated ChIP-chip log ratio is greater than zero. This way, a total of 16 synthetic ChIP-chip datasets were generated for four TFs with various sequence and binding motif lengths. The datasets provide a good opportunity to access the performance of the newly developed BayesPI because we have an expected answer for each synthetic ChIP-chip dataset.

### Microarray datasets

Genome-wide *in vivo *protein-DNA interaction datasets of 203 yeast *S. cerevisiae *TFs and the corresponding intergenic DNA sequences were downloaded from a work by Harbison *et al*. [27], in which the ChIP-chip experiments were performed under various conditions such as rich medium and heat shock, etc. Genome-wide yeast nucleosome occupancy data was obtained from ChIP-chip experiments by Lee *et al*. [68]. Protein expression microarray data of yeast under rich medium conditions were available [39]. ChIP-chip datasets for STE12 and TEC1 in yeasts *S. cerevisiae, S. mikatae*, and *S. bayanus *under pseudohyphal conditions were obtained from a previous work [28]. More information about the pre-processing of these microarray datasets is provided in [Additional file 1].

### Sequence specificity information of yeast DNA-binding proteins

The most recent yeast sequence specificities (~124 TFs) were provided by MacIsaac *et al*. [34]. The experimentally observed sequence specificity information of yeast TFs (~41 TFs) was taken from the TRANSFAC[33] database version 8.3. The consensus sequences of yeast TFs were found in the SGD[65] database (~61 TFs). The position-specific affinity matrices (~203 TFs) inferred by MatrixREDUCE were downloaded from the TransfactomeDB database[32].

### Predicted TF binding parameters in yeast under rich medium conditions

The minimal binding energies (consensus) of 61 yeast TFs in rich medium conditions were generated by both Quadratic Programming Method of Energy Matrix Estimation[21] (QPMEME or QP) and the classical work of Berg and von Hippel[15] (BvH), but the corresponding chemical potentials were provided only by QPMEME. These results were obtained from a work by Aurell *et al*. [26].

### Selection of 61 yeast TFs for the present study

BayesPI requires a full inverse of Hessian matrice which needs compute-intensive calculation. Such computational constraint is one of reasons that we did not consider all available yeast TFs in[27]. Additionally, there are two major quantitative variables that control the binding of TFs to DNA: the cellular abundance and the affinity. The localization of TF proteins in nucleus under the rich medium conditions is a key point to determine the protein activity and its concentration (chemical potential). So far we only found the protein abundance of 61 of yeast TFs could be measured in nucleus [39]. A detailed description of criteria for selecting these TFs are available in paper[26]. Thus, in this work, we only estimated protein binding parameters and protein energy matrices of 61 yeast TFs.

## Competing interests

The authors declare that they have no competing interests.

## Authors' contributions

JW conceived and designed the study, implemented MATLAB program in BayesPI, performed data analysis and results interpretation, and drafted manuscript. MO provided biology consultation and contributed to the manuscript writing. All authors have read and approved the manuscript.

## Supplementary Material

Additional file 1 **Supplementary information of BayesPI - a new model to study protein-DNA interactions**. The file contains support information of BayesPI algorithm, pre-processing of microarray datasets, results of analyzing human ChIP-Seq and other supplementary results etc. Click here for file
