# Supplementary material for: BayesPI - a new model to study protein-DNA interactions: a case study of condition-specific protein binding parameters for Yeast transcription factors
Source: BMC Bioinformatics. 2009 Oct 20;10:345. doi: 10.1186/1471-2105-10-345 (PMC2771022; doi:10.1186/1471-2105-10-345)
Supplement: Additional file 1 — Supplementary information of BayesPI - a new model to study protein-DNA interactions. The file contains support information of BayesPI algorithm, pre-processing of microarray datasets, results of analyzing human ChIP-Seq and other supplementary results etc. [file 1471-2105-10-345-S1.DOC]

**BayesPI - a new model to study protein-DNA interactions: a case study of condition-specific protein binding parameters for Yeast transcription factors**

Junbai Wang and Morigen

The Norwegian Radium Hospital, Rikshospitalet University Hospital

Montebello 0310 Oslo, Norway

**Supplementary Methods**

**Two levels inference of Bayesian Minimization model**

**i) The first level inference: a neural networks implementation for finding the maximum log posterior probability --.** Here we interpret the parameters learning as two-layer neural networks with a fixed non-linear first layer (one neuron) and adaptive linear second layer (one neuron) where back-propagating learning procedure[1] is implemented. For example, if the objective function is

the second layer function is

and the first layer function is

then the derivatives of the second layer parameters $w_{2}$and $b_{2}$ are

and the derivative of the first layer parameters $w_{1}$and $b_{1}$are

where [$w_{1}$, $b_{1}$ $w_{2}$, $b_{2}$] are the model parameters $w_{\text{MP}}$that satisfy the maximum of log posterior probability. After differentiating the log posterior probability with respect to the model parameters of$w$, set the derivatives to zero and apply a scaled conjugate gradient algorithm[2] to find the most probable values of$w_{\text{MP}}$. [3]

**ii) The second level inference: a detailed description of updating evidence values (i.e. $\alpha$ and$\beta$).** Here we first update model parameters of $w_{\text{MP}}$ then infer α and β through Bayes’ rule:

Where is the posterior probability of hyperparameter α and β given the input data D and hypothesis space [A,η,R]. In above equation, the data-dependent term  is the evidence for [α,β] which appears as the normalizing constant in the first level inference. The second term $P(\alpha,\beta|A,\eta,R)$ is the subjective prior over our hypothesis space which expresses how plausible we thought the alternative models were before the data arrived. Here we assume equal priors $P(\alpha,\beta|A,\eta,R)$to the alternative models, thus model [α,β] are ranked only by evaluating the evidence:

This equation has not been normalized by $P(D|A,R,\eta)$because in the data modelling process we may develop new models after the data arrived such as that an inadequacy of the first models is detected. Therefore, the second inference is open ended. We continually seek more probable models to account for the data we gather, and the key point of the second inference is that a Bayesian evaluates the evidence which is the normalizing constant for the posterior probability equation in the first level inference. In other words

or

Since at the first level inference, we approximated the posterior probability distribution

by a Gaussian approximation

Thus the evidence for α and β can be written as:

where $Z_{M}^{'}(\alpha,\beta)$or $Z_{M}^{'}(w_{\text{MP}})$has a single minimum as a function of w, at $w_{\text{MP}}$, after assuming that we can locally approximate M(w) as quadratic there, then the integral $Z_{M}^{'}$ is approximated by a Gaussian integral:

That is

$A=\nabla\nabla M$is the Hessian of M evaluated at $w_{\text{MP}}$ and k is the dimension of w. Therefore, the log evidence of α and β is

Since equations E_D_ and E_w_ are defined by simple quadric functions and the degree of freedom in the data set is N, we can evaluate the Gaussian integrals Z_D_ and Z_w_:

and

Now the log evidence is transformed to:

To find the condition that is satisfied at the maximum of log evidence, we differentiate the log evidence with respect to α:

Since and $\nabla\nabla E_{w}=I$is the identity

And setting the derivative to zero, we obtain the below condition for the most probable value of α

Then we differentiate the log evidence with respect to β:

Thus

Setting the derivative to zero then the condition for the most probable value of β is

If we let

and

Then the maximum evidence values of α and β satisfies:

**iii) Derivation of R-propagation formulas for computing Hessian matrices.** Above two equations can be used as re-estimation formulae for α and β. However, there is an important issue in the calculation, evaluating the Hessian matrix **A**. Here we use an efficient algorithm[4], fast exact multiplication by the Hessian, to compute products **A*v*** without explicitly evaluating **A,** where ***v*** is an arbitrary row vector whose length equals the number of parameters in the neural networks. To calculate **A*v***, we first define a differential operator based on R-propagation algorithm of Pearlmutter[4]

that infers and$R\left\{ w \right\}=v$. Then we apply the operator on simple Back-propagation networks, where error function is

and a_2_ is the network output node and t is the target data.

The forward computation of the network for output node is

hidden node is

input node is

Backward pass is then

R-forward computation is

R-backward computation is

Following above R-back-propagation procedures, we can estimate,, and which are equivalent to compute the Hessian matrix multiplied by an arbitrary vector *v* such as , , and . In above equations, the topology of the neural network sometimes results in some R-variables being guaranteed to be zero when *v* is sparse, and in particular, when vector *v* = (0 … 0 1 0 … 0), which can be used to compute a single desired column of the Hessian matrix A.

**Supplementary Data Analysis**

***Pre-processing of microarray data.*** In this work, we used all available raw ChIP-chip ratios of each TF (~6000 probes in yeast) as the input data to our program without any performance of further data processing and data selecting by p-value. Because low affinity protein binding sites may be functionally important[5]. However, for high resolution yeast tiling array (STE12 and TEC1 in three yeast species[6]) and human ChIP-Seq data[7], we only applied our program on the top 30 percent of available probes (ranked by ratios) and the identified putative TF binding sites (~6000 to ~75000 probes for each of (CTCF, NRSF and STAT1) three human TFs) respectively. This is because of the memory restriction in the computer, which does not allow us to load more than two hundreds thousands probes.

***Motif similarity score.*** *In silico* study of TF binding sites, we often encounter with either the quality evaluation of estimated sequence specificity or the identification of original predicted sequence specificity. Thus, we need a method to compute the similarity between a predicted TF binding motif and a set of known sequence specificity information such as the consensus sequences from the SGD database [8]. In this work, we propose a simple strategy to accomplish the goal.

i) First various representations of TF binding motifs are converted to a common position-specific probability matrix (probabilities of nucleotide i at position j in the position weight matrix[9])

where$f_{i,j}$is the frequency of residue i at position j, $P_{i}$ is the prior frequency for residue i such as (A,0.31; C,0.19; G,0.19; T,0.31)[9] in the yeast genome, and k is the number of k-mers. For example, for SGD consensus motif, we transform it into a relative frequency matrix[10] before converting it to the probabilities. For position-specific energy matrix (the output E_ij_ and ΔG_ij_ of BayesPI), we convert it to$w_{\text{ij}}$ [11, 12] by equation before transforming it into the probabilities[10].

ii) After converting several representations of TF binding motifs (outputs from MatrixREDUCE[13] and MacIsaac *et al.* [14]) to a common position-specific probability matrix, we use an unbiased motif similarity score to perform a fair comparison among various predictions. For example, we compute the similarity between two position-specific probability matrices by aligning the two matrices to maximize a score that defined by Tsai *et al*.[9]

where m is the motif length, w is the number of positions matched between two position-specific probability matrices, and$P(a)_{i,L}$ and $P(b)_{i,L}$ are probabilities of base L at position i in position-specific probability matrix a and b, respectively. For the alignment of two matrices, we consider both forward and reverse DNA sequence, while allow 10% of misalignment between the two position probability matrixes. At the end, the motif with the maximum similarity score is selected as the right answer.

***Analysis of ChIP-Seq experiments using BayesPI.*** Here we first process the raw ChIP-Seq data with SISSRs method[7] to collect a set of putative protein binding sites from short sequence reads (tags) generated by the ChIP-Seq experiment. Then we assume that the tag densities at the binding sites are equivalent to protein-DNA binding affinity. By including these putative protein binding sites and the corresponding tag densities in BayesPI, we may be able to identify protein binding energy matrices within the inferred binding sites.

***Prediction of TF energy matrix by using BayesPI.*** To predict the putative TF energy matrix for either synthetic or real microarray dataset, we defined the minimum length of a TF binding site as its SGD consensus sequence and a maximum length which is five bp longer than the minimum one in the program. However, if there are more than ten hundred thousands input probes (tiling array data of three yeast species[6] and human ChIP-Seq data[7]) then we set the maximum motif length equals to its minimum length due to the concern of compute speed. Then for each input dataset, the program recorded the top six energy matrices which were converted into the position-specific probability matrices before they were compared against the corresponding SGD consensus sequence. Finally, an energy matrix with the maximum motif similarity score was chosen as the putative TF binding energy matrix to the dataset.

***Definition of a reasonable match between two estimates of the protein binding parameters.*** In order to quantify the similarity of protein binding parameters computed by different methods, we first used coefficient of variation (CV) score to screen TFs with reasonable matches between two estimates. For example, two estimated quantities (minimal binding energies by BP and QPMEME) of the same TF have CV<30%, then we define them as a reasonable match. Subsequently, we calculated percentage of TFs that passed the threshold in each pair-wise comparison, and illustrated the scatter plots and the correlation coefficients of these good estimates in the main text (Figure 2). Here we did not directly apply correlation coefficient on all 61 TFs because correlation coefficients measure the strength of a relationship between two variables, not the agreement between them. Data with poor agreement can produce very high correlations[15] and outliers in the data but it will make the correlation coefficients meaningless. This was the case in our study where the predicted protein binding parameters from majority of TFs were close to each other between pair of methods but few of them were extremely different.

**Supplementary Results**

BayesPI program, the estimated position-specific energy matrices with corresponding protein binding parameters for 61 yeast TFs in rich medium conditions, stress conditions, and three yeast species and three human TFs are available on the web [http://www.uio.no/~junbaiw/BayesPI](http://www.uio.no/~junbaiw/bayesianReduce).

***Literature evidences for adaptive modification of protein binding parameters.*** Here we list some of possible literature evidences in supporting of our predicted hypothesis “adaptive modification of protein binding parameters (i.e. protein binding energies) may play roles in the formation of the environment-specific yeast TF binding patterns and in the divergence of TF binding sites across different yeast species”: I) In a classic paper by Fields *et al.* [12] *in vitro* experiments demonstrated that variations of a few key positions in protein (Mnt repressor) binding sites strongly affected its binding or binding energy. Similar evidences can also be found in other publications [16, 17]; particularly, in an excellent review paper[18], strong evidences for binding energy variations were found to associate with nucleotide variation in either binding site or flanking site sequences were shown. II) More recent works have found experimentally and computationally predicted evidences that binding site variations (i.e. weak or strong protein binding) are not only condition dependent but also function specific [19-22]. III) As regard to adaptive evolution of protein binding sties, many papers have been published[23], [24], [25] and [26]; particularly, a study on pure DNA sequences across four yeast species has suggested that there are position specific variations in the rate of evolution in protein binding sites [27]. Thus, we think the present hypothesis is biologically sound.

**Application of the BayesPI on human ChIP-Seq datasets**

Recently, chromatin immunoprecipitation followed by massively paralleled sequencing (ChIP-Seq) has been widely used to investigate genome-wide protein-DNA interactions[7] because the ChIP-Seq experiment produces high-resolution data and avoids several biases that accompany ChIP-chip experiments (i.e., array probe-specific behavior and dye bias[28]) . Here we tried BayesPI on a set of ChIP-Seq datasets [7] for human transcription factors (i.e., CTCF, NRSF and STAT1). The previously identified putative binding sites (26814, 5813 and 73956 for CTCF, NRSF and STAT1 protein, respectively) and the corresponding tag densities at the binding sites were used to infer protein binding energy matrices. The results are encouraging and reveal that our predicted energy matrix of CTCF and STAT1 closely resembled known binding sites although that of NRSF had a weak similarity to the earlier result [7] (SFigure 3, and STable 3). It indicates BayesPI may be a powerful tool to study ChIP-Seq data. Particularly, the application of BayesPI on ChIP-Seq data may avoid several pitfalls (i.e., sequence background model and motif gaps) that accompany multiple sequences alignment algorithms (a common method to identify statistically overrepresented consensus motifs within the inferred binding sties after ChIP-Seq experiment). For example, for estimating the position-specific scoring matrices of three human TFs, we (by BayesPI) utilized all available putative protein binding sites but previous publications (multiple sequence alignment) [7] only used ~5 to ~20% of those putative binding sites.

**Supplementary Figures**

**Figure S1 Distribution of the synthetic ChIP-chip data.** The synthetic DNA sequences were generated by Monte Carlo sampling method through the MATLAB Bay Net toolbox; the corresponding synthetic log ChIP-chip ratios were produced by the MATLAB build-in random number generator. The distribution of synthetic log ChIP-chip ratios (a normal distribution) for 100 random genes in a synthetic SWI4 ChIP-chip experiment is illustrated. Here a SWI4 binding motif was randomly positioned into a synthetic DNA sequence in which the associated log ratio is greater than zero. Then BayesPI program can be directly applied on above dataset to search for the implanted motifs (e.g. SWI4). Both the demo datasets and the MATLAB programs for generating the synthetic ChIP-chip data are included in BayesPI toolbox.

**
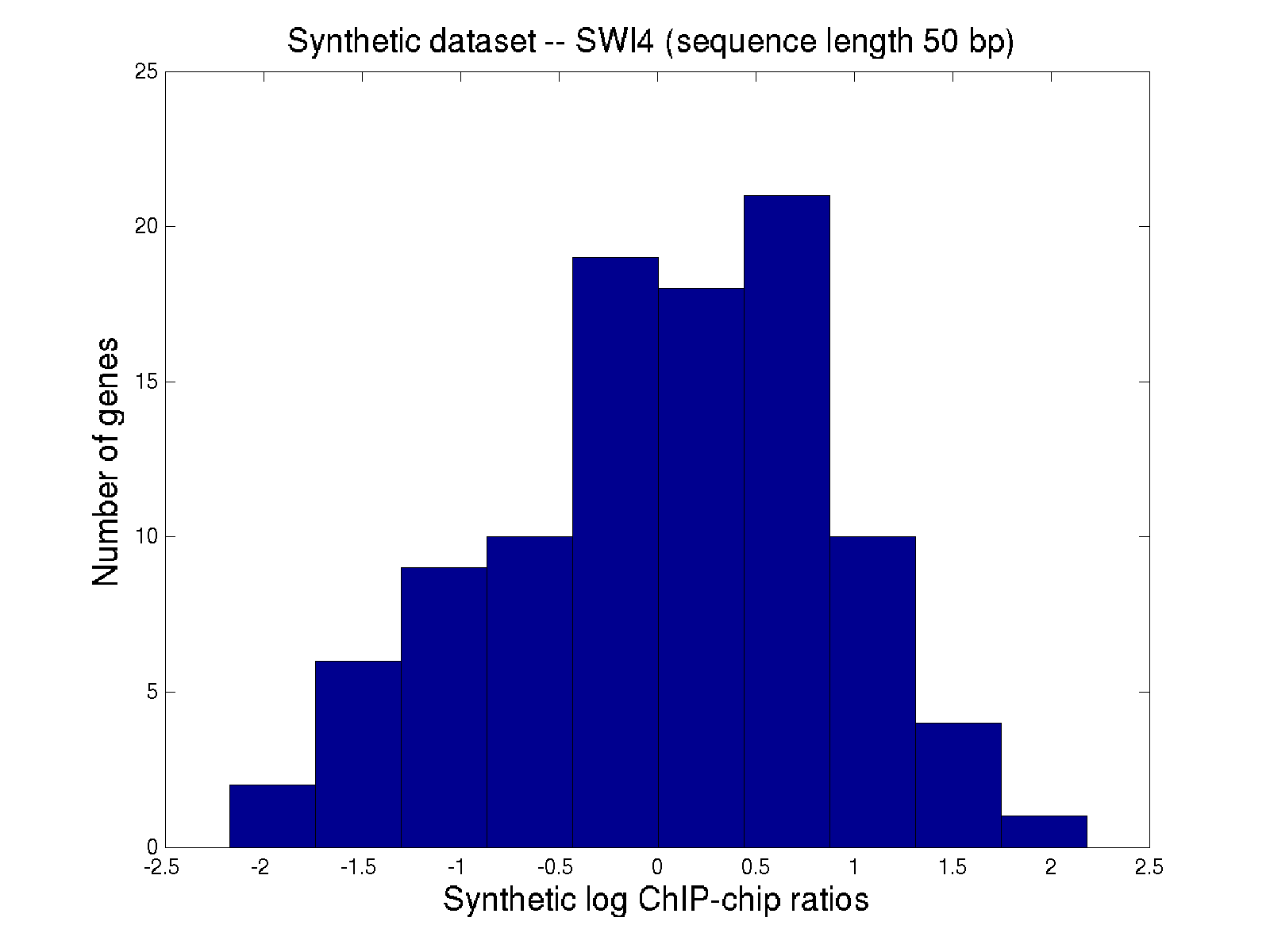
**

**Figure S2. Definition of good matches by using motif similarity score.** To find a pair of motifs that have a reasonable match, we suggest the motif similarity score (MSS) should be greater than 0.75. The reason of using such cutoff value can be explained by a simple scatter plot shown here, in which MSS from 16 synthetic datasets are plotted against their corresponding percentage of binding sites matched to SGD consensus sequence (PBSM). The plot shows that there is a critical value for MSS: if MSS>0.75 then almost all of the PBSM are greater than 0.6 except for one. A detailed study of motif similarity score and its real application can be found in earlier publications [9, 29], where the same score cutoff value was used and MSS was shown to be a robust method to quantify the similarity between a pair of motifs.

**
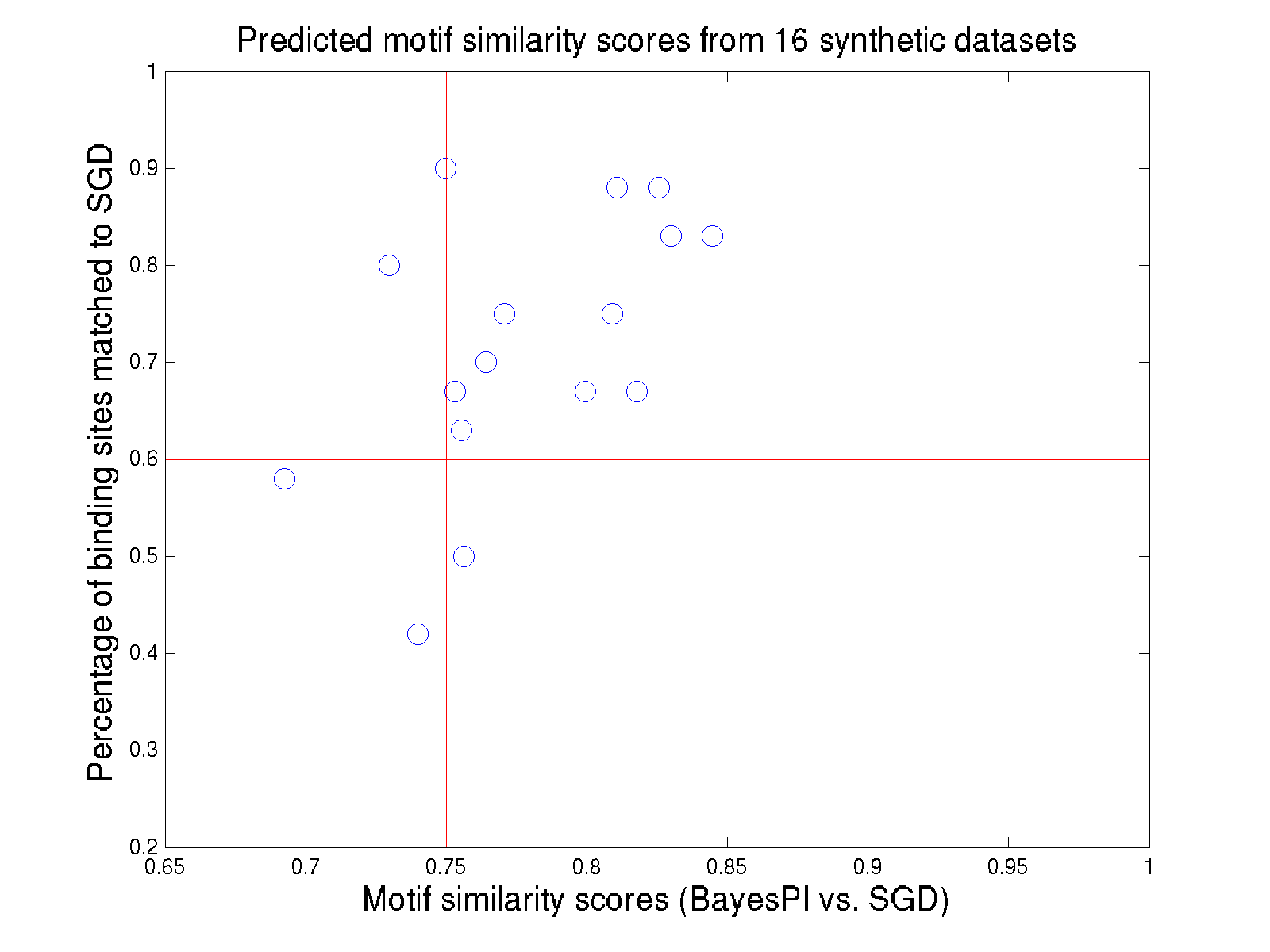
**

**Figure S3 Predicted protein binding energy matrices for three human TFs.** The energy matrices of human TFs are estimated by the BayesPI using ChIP-Seq data[7]. The sequence logo was generated by energy matrices by the BayesPI. Here we used previously identified putative binding sites (~26814 probes to CTCF, ~5813 to NRSF and ~73956 to STAT1) as input data to the BayesPI. To compute the energy matrices, four different motif lengths were chosen for NRSF but only one motif length was selected for CTCF and STAT1 in the program.

**
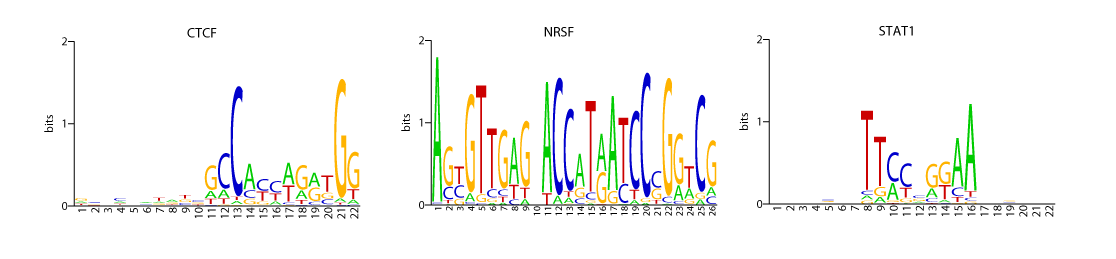
**

**Figure S4 Species-specific binding energy matrices for yeast STE12.** STE12 binding energy matrices were estimated by the BayesPI using ChIP-chip experimental data from *S. cerevisiae* (Scer), *S. mikatae* (Smik), and *S. bayanus* (Sbay) under pseudohyphal conditions. R represents replicated experiment, D means dye swapped experiment and the STE12 binding site (TGAAACR) is underlined in black. The sequence logo was generated by the energy matrices estimated by the BayesPI.

**
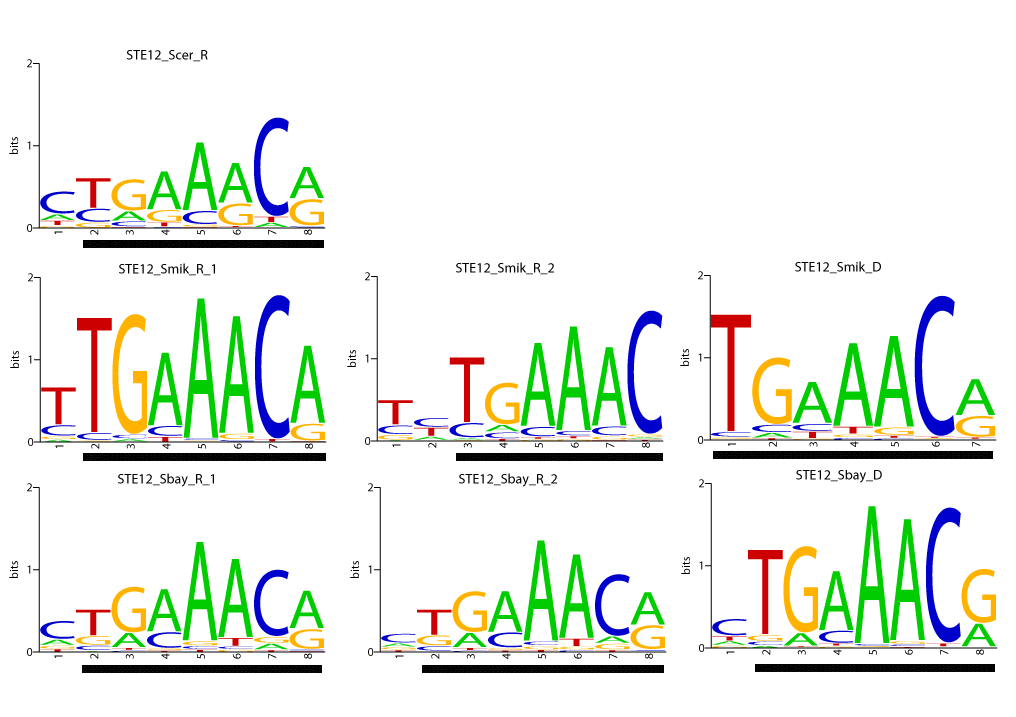
**

**Figure S5 Species-specific binding energy matrices for yeast TEC1.** TEC1 binding energy matrices were estimated by the BayesPI using ChIP-chip experimental data from *S. cerevisiae* (Scer), *S. mikatae* (Smik), and *S. bayanus* (Sbay) under pseudohyphal conditions. R represents replicated experiment, D means dye swapped experiment and the TEC1 binding site (CATTCY) is underlined in black. The sequence logo was generated by the energy matrices estimated by the BayesPI.

**
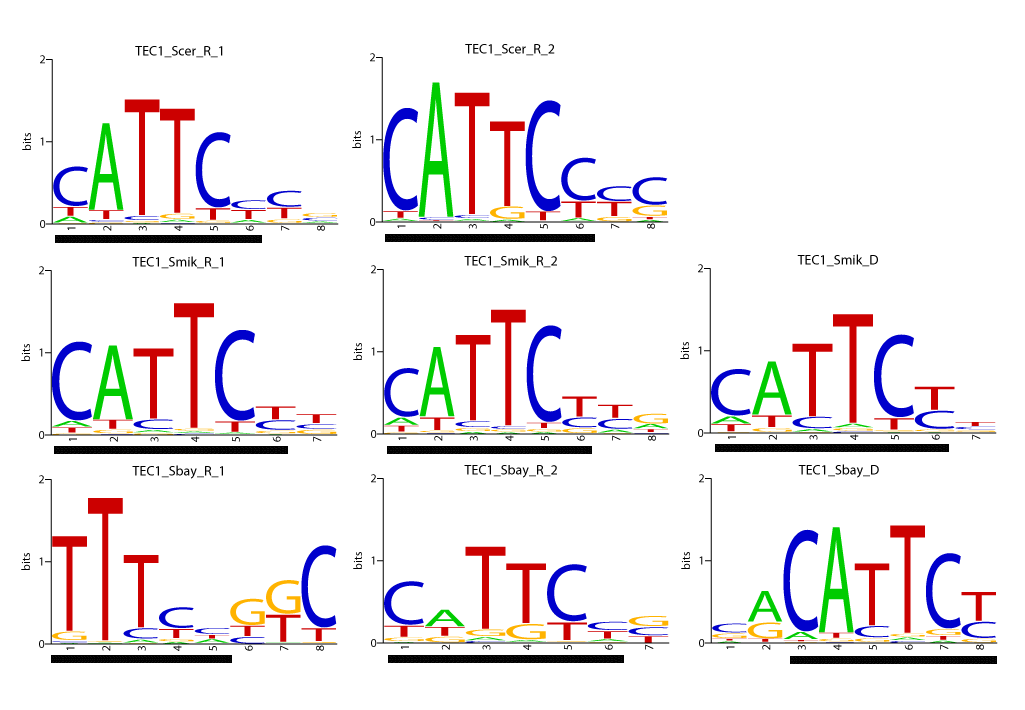
**

**Supplementary Tables**

**Table S1 Comparison of binding parameters for a set of 61 TFs of the yeast *S. cerevisiae* (YPD condition).**

| **TF** |  |  |  |  |  |
| --- | --- | --- | --- | --- | --- |
| abf1 | -25.10 | 20.53 | -15.11 | 12.86 | -30.10 |
| ace2 | -16.37 | 17.16 | -17.63 | 17.63 | -12.65 |
| bas1 | -23.25 | 23.14 | -23.25 | 23.25 | -15.21 |
| cad1 | -36.47 | 32.48 | -19.35 | 17.51 | -14.44 |
| dig1 | -18.92 | 20.08 | - | - | -16.37 |
| fhl1 | -45.88 | 34.83 | -25.08 | 20.60 | -26.41 |
| fkh1 | -21.46 | 22.47 | - | - | -19.71 |
| fkh2 | -19.80 | 18.49 | - | - | -23.54 |
| gal4 | -16.30 | 13.34 | -19.29 | 15.89 | -20.41 |
| gal80 | -15.09 | 10.24 | -12.59 | 12.28 | -15.10 |
| gcr1 | -19.13 | 20.27 | -21.36 | 18.83 | -12.34 |
| gln3 | -25.41 | 20.56 | - | - | -18.08 |
| hap5 | -14.26 | 12.75 | - | - | -11.57 |
| ino2 | -25.89 | 23.94 | - | - | -15.26 |
| ino4 | -18.56 | 15.42 | -41.01 | 34.08 | -18.95 |
| leu3 | -21.33 | 11.63 | -20.37 | 18.42 | -15.24 |
| mac1 | -27.04 | 28.17 | -10.98 | 10.71 | -8.60 |
| mbp1 | -18.29 | 19.51 | - | - | -20.12 |
| mcm1 | -43.30 | 29.20 | -13.67 | 11.06 | -26.07 |
| met31 | -16.29 | 12.33 | -16.14 | 16.14 | -10.87 |
| met4 | -34.74 | 22.03 | -14.02 | 12.34 | -15.96 |
| mot3 | -22.13 | 21.65 | - | - | -9.25 |
| msn2 | -21.96 | 13.97 | - | - | -14.10 |
| ndd1 | -32.44 | 25.57 | -18.44 | 16.10 | -21.42 |
| nrg1 | -19.75 | 13.59 | - | - | -17.68 |
| pdr1 | -18.01 | 13.08 | -12.93 | 12.51 | -7.01 |
| pdr3 | -25.05 | 24.42 | - | - | -5.18 |
| phd1 | -14.28 | 15.10 | - | - | -15.45 |
| pho2 | -19.41 | 18.59 | -11.37 | 10.40 | -8.97 |
| put3 | -19.92 | 15.06 | -12.49 | 11.85 | -15.96 |
| rap1 | -25.53 | 15.43 | -18.31 | 14.60 | -23.09 |
| rcs1 | -16.66 | 12.51 | -24.37 | 21.04 | -16.85 |
| reb1 | -18.10 | 18.25 | -15.12 | 13.02 | -23.68 |
| rfx1 | -30.64 | 25.44 | -16.66 | 14.97 | -18.44 |
| rgt1 | -19.45 | 14.31 | - | - | -12.58 |
| rlm1 | -27.60 | 22.16 | -27.54 | 24.46 | -14.40 |
| rlr1 | -19.40 | 16.82 | -20.81 | 19.66 | -11.10 |
| rox1 | -22.71 | 14.83 | - | - | -13.49 |
| rtg3 | -10.63 | 8.57 | -15.63 | 15.63 | -7.43 |
| sfp1 | -21.43 | 19.81 | -56.71 | 46.90 | -17.81 |
| skn7 | -15.70 | 15.09 | -25.77 | 20.63 | -18.55 |
| sko1 | -21.12 | 19.14 | -22.35 | 22.35 | -9.89 |
| sok2 | -17.37 | 16.75 | - | - | -16.16 |
| spt2 | -22.53 | 20.15 | - | - | -16.26 |
| spt23 | -19.93 | 13.95 | - | - | -13.00 |
| stb1 | -18.30 | 17.58 | -31.10 | 27.00 | -17.94 |
| stb4 | -11.93 | 9.78 | -18.70 | 18.69 | -10.11 |
| stb5 | -21.97 | 20.69 | -28.06 | 25.07 | -16.32 |
| ste12 | -17.80 | 16.40 | - | - | -18.19 |
| sum1 | -22.59 | 19.22 | - | - | -20.65 |
| swi4 | -28.88 | 20.67 | - | - | -21.42 |
| swi5 | -14.66 | 14.40 | -37.53 | 37.53 | -14.88 |
| swi6 | -31.19 | 24.73 | -32.95 | 26.45 | -19.93 |
| tec1 | -20.83 | 20.27 | - | - | -15.84 |
| tye7 | -24.95 | 20.37 | -20.86 | 19.15 | -18.00 |
| ume6 | -19.07 | 16.85 | -30.04 | 24.83 | -26.54 |
| xbp1 | -20.60 | 12.94 | -19.74 | 19.74 | -5.83 |
| yap1 | -22.63 | 21.38 | -19.66 | 18.47 | -13.73 |
| yap6 | -22.22 | 22.23 | -19.51 | 19.51 | -6.87 |
| yap7 | -18.29 | 16.15 | - | - | -20.14 |
| yox1 | -15.81 | 10.44 | -19.11 | 17.42 | -10.67 |

$E_{\text{BP}}$ and are minimal binding energy (consensus) and absolute chemical potential estimated by the BayesPI, $E_{\text{QP}}$ and are minimal binding energy (consensus) and absolute chemical potential obtained by QPMEME[30], and $E_{\text{BvH}}$ is the minimal binding energy (consensus) computed from BvH[10]. The chemical potential is given as a unit of RT.

**Table S2 A comparison of the predicted TF energy matrices between with and without nucleosome positioning information by the BayesPI.**

| TF name | **Without nucleosome positioning** | | | | **With nucleosome positioning** | | | |
| --- | --- | --- | --- | --- | --- | --- | --- | --- |
| *order* | *score* | *μ* | *E* | *order* | *Score* | *μ* | *E* |
| met31 | 0 | 0.74 | -12.33 | -16.29 | 0 | 0.73 | -10.05 | -11.61 |
| rfx1 | 0 | 0.66 | -25.44 | -30.64 | 0 | 0.67 | -31.03 | -42.58 |
| pdr1 | 0 | **0.74** | -13.08 | -18.01 | 6 | **0.78** | -13.37 | -18.94 |
| swi4 | 1 | **0.78** | -20.67 | -28.88 | 1 | **0.80** | -43.02 | -57.33 |
| ace2 | **6** | 0.94 | -17.16 | -16.37 | **5** | 0.90 | -22.68 | -25.44 |
| abf1 | 2 | 0.91 | -20.53 | -25.10 | 2 | 0.91 | -20.53 | -25.08 |
| mbp1 | **3** | 0.92 | -19.51 | -18.29 | **1** | 0.91 | -17.96 | -17.12 |
| rap1 | 1 | 0.88 | -15.43 | -25.53 | 2 | 0.82 | -21.37 | -31.57 |
| leu3 | **3** | 0.76 | -11.63 | -21.33 | **2** | 0.79 | -10.95 | -18.55 |
| mcm1 | 1 | **0.79** | -29.20 | -43.30 | 6 | **0.86** | -28.11 | -40.92 |

Here three (Met31, Rfx1 and Pdr1) of 10 TFs are not functional under rich medium conditions but the rest seven are active in the rich medium conditions. *order* represents rank order of the best predicted motif, *score* is the motif similarity score when the predicted energy matrices are compared with the SGD consensus motif (if score is <0.75 then the order is 0 because the best motif has poor quality), *μ* is the chemical potential and *E* is the protein minimal binding energy.

**Table S3 Motif similarity scores of predicted human TF energy matrices (three TFs in SFigure 3) versus the known consensus sequences (or weight matrices from JASPAR).**

| Name | tggccasyagrkggcrsyr (CTCF) | TTCAGCACCA (NRSF) | TTTCCYRKAA (STAT1) | STAT1 (JASPAR) |
| --- | --- | --- | --- | --- |
| CTCF | **0.78** |  |  |  |
| NRSF |  | **0.79** |  |  |
| STAT1 |  |  | **0.78** |  |
| STAT1 |  |  |  | 0.60 |

In JASPAR[31] database, we only find one (STAT1) of the three TFs. Consensus sequences of three human TFs are obtained from an earlier publication[7].

**Supplementary References**

1. Rumelhart D.E., Hinton G.E., R.J. W: **Learning representations by back-progagating errors**. *Nature* 1986, **323**(9).

2. Møller ME: **A scaled conjugate gradient algorithm for fast supervised learning**. *Neural Networks* 1993, **6**(4).

3. Mackay D: **Bayesian Methods for Adaptive Models**. *PhD thesis, California Institute of Technology* 1991.

4. Pearlmutter BA: **Fast exact multiplication by the Hessian**. *Neural Computation* 1994, **6**(1).

5. Tanay A: **Extensive low-affinity transcriptional interactions in the yeast genome**. *Genome research* 2006, **16**(8):962-972.

6. Borneman AR, Gianoulis TA, Zhang ZD, Yu H, Rozowsky J, Seringhaus MR, Wang LY, Gerstein M, Snyder M: **Divergence of transcription factor binding sites across related yeast species**. *Science (New York, NY* 2007, **317**(5839):815-819.

7. Jothi R, Cuddapah S, Barski A, Cui K, Zhao K: **Genome-wide identification of in vivo protein-DNA binding sites from ChIP-Seq data**. *Nucleic acids research* 2008, **36**(16):5221-5231.

8. Cherry JM, Adler C, Ball C, Chervitz SA, Dwight SS, Hester ET, Jia Y, Juvik G, Roe T, Schroeder M *et al*: **SGD: Saccharomyces Genome Database**. *Nucleic acids research* 1998, **26**(1):73-79.

9. Tsai HK, Huang GT, Chou MY, Lu HH, Li WH: **Method for identifying transcription factor binding sites in yeast**. *Bioinformatics (Oxford, England)* 2006, **22**(14):1675-1681.

10. Berg OG, von Hippel PH: **Selection of DNA binding sites by regulatory proteins. Statistical-mechanical theory and application to operators and promoters**. *Journal of molecular biology* 1987, **193**(4):723-750.

11. Buchler NE, Gerland U, Hwa T: **On schemes of combinatorial transcription logic**. *Proceedings of the National Academy of Sciences of the United States of America* 2003, **100**(9):5136-5141.

12. Fields DS, He Y, Al-Uzri AY, Stormo GD: **Quantitative specificity of the Mnt repressor**. *Journal of molecular biology* 1997, **271**(2):178-194.

13. Foat BC, Tepper RG, Bussemaker HJ: **TransfactomeDB: a resource for exploring the nucleotide sequence specificity and condition-specific regulatory activity of trans-acting factors**. *Nucleic acids research* 2008, **36**(Database issue):D125-131.

14. MacIsaac KD, Wang T, Gordon DB, Gifford DK, Stormo GD, Fraenkel E: **An improved map of conserved regulatory sites for Saccharomyces cerevisiae**. *BMC bioinformatics* 2006, **7**:113.

15. Bland JM, Altman DG: **Statistical methods for assessing agreement between two methods of clinical measurement**. *Lancet* 1986, **1**(8476):307-310.

16. Man TK, Stormo GD: **Non-independence of Mnt repressor-operator interaction determined by a new quantitative multiple fluorescence relative affinity (QuMFRA) assay**. *Nucleic acids research* 2001, **29**(12):2471-2478.

17. Liu X, Clarke ND: **Rationalization of gene regulation by a eukaryotic transcription factor: calculation of regulatory region occupancy from predicted binding affinities**. *Journal of molecular biology* 2002, **323**(1):1-8.

18. Jen-Jacobson L: **Protein-DNA recognition complexes: conservation of structure and binding energy in the transition state**. *Biopolymers* 1997, **44**(2):153-180.

19. Bond GL, Hu W, Levine A: **A single nucleotide polymorphism in the MDM2 gene: from a molecular and cellular explanation to clinical effect**. *Cancer research* 2005, **65**(13):5481-5484.

20. Tuteja G, Jensen ST, White P, Kaestner KH: **Cis-regulatory modules in the mammalian liver: composition depends on strength of Foxa2 consensus site**. *Nucleic acids research* 2008, **36**(12):4149-4157.

21. Segal L, Lapidot M, Solan Z, Ruppin E, Pilpel Y, Horn D: **Nucleotide variation of regulatory motifs may lead to distinct expression patterns**. *Bioinformatics (Oxford, England)* 2007, **23**(13):i440-449.

22. Buck MJ, Lieb JD: **A chromatin-mediated mechanism for specification of conditional transcription factor targets**. *Nature genetics* 2006, **38**(12):1446-1451.

23. Tsong AE, Tuch BB, Li H, Johnson AD: **Evolution of alternative transcriptional circuits with identical logic**. *Nature* 2006, **443**(7110):415-420.

24. Lynch VJ, Tanzer A, Wang Y, Leung FC, Gellersen B, Emera D, Wagner GP: **Adaptive changes in the transcription factor HoxA-11 are essential for the evolution of pregnancy in mammals**. *Proceedings of the National Academy of Sciences of the United States of America* 2008, **105**(39):14928-14933.

25. Mustonen V, Kinney J, Callan CG, Jr., Lassig M: **Energy-dependent fitness: a quantitative model for the evolution of yeast transcription factor binding sites**. *Proceedings of the National Academy of Sciences of the United States of America* 2008, **105**(34):12376-12381.

26. Berg J, Willmann S, Lassig M: **Adaptive evolution of transcription factor binding sites**. *BMC evolutionary biology* 2004, **4**(1):42.

27. Moses AM, Chiang DY, Kellis M, Lander ES, Eisen MB: **Position specific variation in the rate of evolution in transcription factor binding sites**. *BMC evolutionary biology* 2003, **3**:19.

28. Qi Y, Rolfe A, MacIsaac KD, Gerber GK, Pokholok D, Zeitlinger J, Danford T, Dowell RD, Fraenkel E, Jaakkola TS *et al*: **High-resolution computational models of genome binding events**. *Nature biotechnology* 2006, **24**(8):963-970.

29. Chen CY, Tsai HK, Hsu CM, May Chen MJ, Hung HG, Huang GT, Li WH: **Discovering gapped binding sites of yeast transcription factors**. *Proceedings of the National Academy of Sciences of the United States of America* 2008, **105**(7):2527-2532.

30. Djordjevic M, Sengupta AM, Shraiman BI: **A biophysical approach to transcription factor binding site discovery**. *Genome research* 2003, **13**(11):2381-2390.

31. Bryne JC, Valen E, Tang MH, Marstrand T, Winther O, da Piedade I, Krogh A, Lenhard B, Sandelin A: **JASPAR, the open access database of transcription factor-binding profiles: new content and tools in the 2008 update**. *Nucleic acids research* 2008, **36**(Database issue):D102-106.
